# Evaluation of the Content Validity and Cross-Cultural Validity of the Study Participant Feedback Questionnaire (SPFQ)

**DOI:** 10.1007/s43441-020-00179-3

**Published:** 2020-07-20

**Authors:** Alison Greene, Mary Elmer, Sean Ludlam, Kathyjo Shay, Sarah Bentley, Claire Trennery, Rebecca Grimes, Adam Gater

**Affiliations:** 1 Patient-Centered Outcomes Research, Genentech, San Francisco, CA 94080 USA; 2 Merck & Co., Inc., Upper Gwynedd, PA 19446 USA; 3 Clinical Records Management, Allergan, Madison, NJ 07940 USA; 4 Clinical Science Center of Excellence, Astellas Pharma Global Development, Inc., Northbrook, IL 60062 USA; 5 Patient-Centered Outcomes, Adelphi Values, Bollington, SK10 5JB UK

**Keywords:** Clinical trial feedback, SPFQ, Patient experience, Qualitative research, Cross-cultural validity

## Abstract

**Objectives:**

The Study Participant Feedback Questionnaire (SPFQ) is a patient-completed tool designed to assess patient experiences and satisfaction with aspects associated with being involved in a clinical trial. Originally developed in oncology and among English-speaking participants, the aim of the current study was to evaluate the content and cross-cultural validity of the SPFQ in other indications and non-English-speaking countries.

**Methods:**

Semi-structured qualitative telephone interviews were conducted with 80 participants across eight non-English-speaking countries (in Europe, South America and Asia) who had received an investigational medicinal product as part of a clinical trial in the past three years. Interviews comprised concept elicitation to identify concepts of importance to participants’ trial experiences, and cognitive debriefing to assess understanding and perceived importance of SPFQ instructions, items and response options.

**Results:**

Concept elicitation findings supported the content validity of the SPFQ. During cognitive debriefing, SPFQ instructions and the majority of items were well understood by participants. Participants generally considered the SPFQ items important to their clinical trial experience, albeit a handful of items assessed concepts that had not been experienced by trial participants or were redundant with other SPFQ items. The instructions, response options and recall period of the SPFQ were generally well understood. No country-level differences in understanding or importance were apparent.

**Conclusion:**

Study findings provide evidence for the content and cross-cultural validity of the SPFQ and support implementation of the SPFQ as a means of obtaining participant feedback across global development programmes in a variety of indications.

**Electronic supplementary material:**

The online version of this article (10.1007/s43441-020-00179-3) contains supplementary material, which is available to authorized users.

## Introduction

The importance of patient engagement in clinical trial design is well established [1–4]. Improved clinical trial design and execution could ultimately lead to a better patient experience [5]. Additionally, designing a clinical trial with procedures that have been confirmed as acceptable to participants is likely to improve patient adherence to treatments and engagement in healthcare [6], and thus the validity of the trial findings. The complexity of clinical trials means that obtaining participant perspectives regarding clinical trials at key points throughout the clinical trial process (i.e. at the beginning/before, during and at the end/after) could be particularly valuable for optimising trial design and implementation.

There are few successful examples of substantive patient contribution to clinical trial design and process, especially while actively participating in the trial [7, 8]. Those instruments that do exist to evaluate participant feedback have generally only been developed and evaluated in a specific trial design or for implementation in one indication [9]. Therefore, there is an unmet need for an instrument that could comprehensively evaluate participant experience in a variety of clinical trials globally, across a range of therapeutic indications, among diverse populations, and at key points throughout a clinical trial [10].

The Study Participant Feedback Questionnaire (SPFQ) was developed as a tool that could be used in clinical trials to collect feedback from participants regarding their trial experience. Originally developed as the Trial Feedback Questionnaire (TFQ) for use in oncology trials in the US and UK [11, 12], in line with best practice approaches [3, 13], the current SPFQ is a patient-completed measure assessing clinical trial participant satisfaction and understanding of clinical trial procedures and experiences across a broad range of indications, and global regions.

The complete SPFQ is comprised of 23 questions (items) assessing patients’ experiences as clinical trial participants. The instrument is split into three separate questionnaires to be completed at different timepoints throughout the trial process: the SPFQ-A (4–6 items) to be administered at enrolment, the SPFQ-B (8–12 items) to be administered to participants during the trial, and the SPFQ-C (5 items) to be administered to participants upon their completion of the trial [12]. Items in the SPFQ assess concepts such as satisfaction with trial procedures, provision and understanding of key information, impact on daily life, ability to ask questions and the quality of the answers provided and support following the trial completion. A key benefit of collecting data at three timepoints across the clinical trial is that feedback can be used to highlight issues and implement changes either as the trial progresses (e.g. addressing recruitment barriers or potential reasons for early withdrawal) or in consideration for future trial protocols. The SPFQ is provided as a supplementary file and is available for full download: https://transceleratebiopharmainc.com/patientexperience/study-participant-feedback-questionnaire/.

For the continued development of the SPFQ, evidence is required to support the use of the instrument across global development programmes in a variety of therapeutic indications. Therefore, the overall objective of this study was to further evaluate the content validity and cross-cultural validity of the SPFQ through the conduct of concept confirmation interviews in a diverse sample of patients recruited primarily from non-English-speaking countries. The interviews aimed to (1) explore the qualitative patient experience of being involved in a clinical trial, specifically to understand concepts of importance relating to trial experience and elicit patient-friendly language relating to these concepts, and (2) cognitively debrief the SPFQ, specifically to assess understanding and perceived importance of all aspects of the instrument including instructions, items, response options and recall periods.

## Methods

This was a non-interventional, qualitative interview study with 80 participants across eight non-English-speaking countries: France, Italy, Germany, Spain, Poland, Brazil, India and China (*n* = 10 participants per country). Countries were selected to ensure geographic, cultural and linguistic diversity within the recruited sample. Sixty-minute combined concept elicitation (CE) and cognitive debriefing (CD) interviews were conducted via telephone which aimed to explore participants’ clinical trial experiences and confirm the content and cross-cultural validity of the SPFQ (see supplementary file). This study was submitted to an independent international ethical board for approval prior to any study related activities and fieldwork being conducted (Salus IRB: TD8149A). Written informed consent was obtained from all participants prior to entry into the study.

### Study Sample

Participants must have been at least 18 years old at the time of recruitment and must have taken part in an interventional (drug or medical device) clinical trial in the past three years for treatment of a health condition. Participants were excluded from the study if they had taken part in a trial as a healthy volunteer or were enrolled in any other trial/research study involving a health condition at the time of screening.

Participants were identified through a third-party agency and were recruited via: consumer and patient databases/panels, physician referral, patient help groups/association postings and previously known trial participants in each country. All participants were provided with an information letter outlining the study requirements and those who were interested in participating completed an electronic information and consent form, indicating their willingness to take part in the study. Once consent was obtained, screening calls were conducted to determine eligibility and collect basic demographic and clinical information such as age, sex, the condition that the participant had been diagnosed with, and the time since the clinical trial.

Target recruitment quotas were set to ensure a diverse sample of participants with respect to age, sex, education level, race, therapeutic area, clinical trial phase, time since participation in clinical trial and whether the participant had withdrawn early from the study. Recruitment was limited to no more than three participants from the same clinical trial (overall) or indication (per country) in order to ensure broad representation.

### Interview Process

Interviews were conducted over the telephone in participants’ native language by trained researchers using a semi-structured interview guide. Evidence suggests that while some information is sacrificed in telephone interviews (e.g. non-verbal communications), little to no data quality is lost when conducting interviews via telephone versus in-person [14]. Each participant (who had not seen the SPFQ before) was provided with a link to a web-based version of the SPFQ in the appropriate language which they were required to access during the interview.

Interviews consisted of a brief concept elicitation section which was dedicated to understanding the participant’s experience of being involved in a clinical trial, including trial aims, treatment processes and motivations for participation. The interviewer utilised open-ended and unbiased questioning to establish concepts that were of greatest importance to participants, as well as identifying language used by participants when describing aspects of their clinical trial. Participants were given an opportunity to highlight their opinions ‘spontaneously’. Following this, participants were asked more focused questions designed to probe on topics that they may not have mentioned.

At the start of the second part of the interview, participants were prompted to open the online link to the SPFQ. Following this, cognitive debriefing of the SPFQ was conducted which required participants to complete the web-based questionnaire using a ‘think-aloud’ technique, followed by in-depth debriefing questioning. Participants were asked to read out each instruction and item before a vocalising their thoughts while selecting an answer. Participants were asked detailed questions about their understanding of the items, response options, instructions and recall period, and were asked to comment on the perceived importance of the items (i.e. the extent to which the participant thought each item assessed a concept that was relevant and important to their overall trial experience). Participants were also asked to provide general feedback on the SPFQ, which evaluated aspects of the questionnaire such as: items for removal/addition, ease of completion, and positive/negative aspects of the questionnaire.

### Qualitative Analysis

All interviews were recorded and transcribed verbatim prior to translation into English language for analysis. All transcripts were dual-forward translated and reconciled before being certified for analysis. All analyses were performed using Atlas Ti. Software (Atlas.Ti Scientific Software Development GmbH, Berlin, Germany) [15]. Transcripts were assessed, coded and analysed by two researchers and reviewed by the study lead, to ensure consistency. Analysis was conducted at a subgroup level to assess any differences between participants based on demographic, geographic and trial characteristics. Thematic analysis methods were utilised whereby sections of transcripts from individual participants (i.e. quotes) were assigned codes reflective of underlying concepts [16–19]. Qualitative analysis of participant responses during cognitive debriefing focused specifically on whether the concepts and items of the SPFQ were understood and accurately interpreted by participants and also important to their clinical trial experience [3]. Instances where items were not understood/consistently interpreted or considered important by ≥ 10% of participants were highlighted.

## Results

### Demographic and Clinical Characteristics

A total of 80 participants took part in this qualitative study (10 participants from each country). The age of the sample ranged from 19–72 with a mean of 45.1 years. The sex of the sample was also evenly distributed, with a total of 39 males (48.7%) and 41 females (51.3%). Most participants described their race as White (*n* = 47/80; 58.8%). The sample also included participants who were Asian (*n* = 21/80; 26.3%) and Multi-racial (*n* = 2/80; 2.5%). The largest proportion of the sample had some higher education (*n* = 35/80; 43.8%) however there was good representation of participants with a high school (only) education (*n* = 24/80; 30.0%).

A total of 17 therapeutic areas were represented. The highest numbers of participants had oncological (*n* = 12/80; 15%) and nutritional/metabolic disorders (*n* = 11/80; 13.8%). Therapeutic areas were well distributed across each country. Approximately one third of the sample each completed their trial within 0–11 months (*n* = 29/80; 36.3%), 12–23 months (*n* = 25/80; 31.3%) and 24–36 months prior to recruitment (*n* = 26/80; 32.5%). A total of six participants who withdraw from their clinical trial early were included in the study sample (*n* = 6/80; 7.5%). Further detail relating to study sample demographic and clinical characteristics can be found in Table 1. Country-specific demographic results are provided as supplementary material.

**Table 1:** Overview of Clinical and Demographic Characteristics.

Characteristic	Total (*n* = 80)
Age	
Range (min–max)	19–72
Mean	45.1
Sex, *n* (%)	
Male	39 (48.7)
Female	41 (51.3)
Education, *n* (%)	
Some years/completed high school	24 (30)
Some years of higher education (college/university)	35 (43.8)
Vocational qualifications	6 (7.5)
Post-graduate qualifications	15 (18.8)
Race, *n* (%)	
White	47 (58.8)
Asian	21 (26)
Multi-racial	2 (2.5)
Not permitted to ask	10 (12.5)
Stage of clinical trial, *n* (%)	
Phase I	3 (3.8)
Phase II	9 (11.3)
Phase III	19 (23.8)
Phase IV	4 (5)
Not available	45 (56.3)
Time since trial completion, *n* (%)	
0–11 months	29 (36.3)
12–23 months	25 (31.3)
24–36 months	26 (32.5)
Trial withdrawers, *n* (%)	
No	74 (92.5)
Yes	6 (7.5)
Therapeutic area, *n* (%)	
Bacterial infection	4 (5)
Cardiovascular	1 (1.3)
Dermatological	6 (7.8)
Digestive	2 (2.6)
Endocrinological	2 (2.6)
Genitourinary	3 (3.9)
Haematological	3 (3.9)
Mental and behavioural	5 (6.5)
Musculoskeletal disorders	8 (10)
Neurological disorders	5 (6.5)
Nutritional/metabolic	11 (13.8)
Oncological	12 (15)
Ophthalmological	1 (1.3)
Otorhinolaryngologic	3 (3.9)
Pathological	1 (1.3)
Respiratory	8 (10)
Viral infection	5 (6.5)

### Concept Elicitation Results

Findings indicated that ‘participants’ was the preferred term to describe those involved in a clinical trial (*n* = 43/77; 55.8%). Motivations for participation included participants trying to improve their health condition (*n* = 43/72; 59.7%), being advised by their doctor (*n* = 16/72; 22.2%), being part of important research (*n* = 15/72; 20.8%) and wanting access to treatments they typically would not be able to access (*n* = 10/72; 13.9%).

Receiving high quality medical care including frequent monitoring (*n* = 31/75; 41.3%), seeing improvements to their condition (*n* = 31/75; 41.3%) and the attitude of trial staff (*n* = 14/75; 18.7%) were the most commonly reported positive aspects of participants’ trial experience. Negative aspects included the time-consuming nature of trials, including the length and number of visits and extensive follow-up (*n* = 15/62; 24.2%) and poor communication with trial staff (*n* = 11/62; 17.7%).

In line with this feedback, participants were also asked to explain how their trial experience could have been improved (*n* = 20/80; 25.0%). These participants reported that they would like both verbal and written communication to be clearer and easier for them to understand (*n* = 7/20; 35.0%). They also suggested that follow-up after the trial about the status of the investigational product, e.g. when will it be available, could be improved (*n* = 5/20; 25.0%). A selection of quotes supporting findings from the concept elicitation section of the interviews can be found in Table 2. Of note, no regional differences were observed upon analysis of the concept elicitation findings.

**Table 2:** Selection of Quotes from Concept Elicitation Interviews.

Trial Feedback	Participant Quote(s)
Motivations for trial participation	“There is, there was a higher chance for successful embryo transfer, if that drug had worked.” (improvement of health condition) “Because I trust in my neurologist, and I had no doubts at all. If the neurologist recommends something, I follow his advice.” (advice from clinical professional) “I knew it was an important study and it could change lives and save people and maybe it could help me as well.” (helping others)
Positive aspects of trial participation	“…normally I would have to pay for medical care. I had some blood tests done, I had my heart examined, had cardiogram done.” (monitoring of health condition) “That I noticed improvements in the long-term, that is, it was more effective.” (improvement of health condition) “So first it was a small team. There were great listeners and very kind.” (attitude of trial staff)
Negatives of trial participation	“It was very frequent I had to follow up once half a month or once a month. I’d like to accept the frequency of once 3 months or once half a year when my condition had become stable.” (time-consuming follow-up) “I often failed to contact to anybody. In some cases, the staff on duty were not clear about some information.” (poor communication with trial staff)
Improvements to trial participation in the future	“There were some papers to fill at the hospital…I remember there were some words quite difficult to understand.” (improved written communication) “Let’s say I could have been contacted…to let me know if the product would arrive on the market.” (improved follow-up relating to investigational product)

### Cognitive Debriefing of the SPFQ

#### SPFQ-A

**Figure 1:**
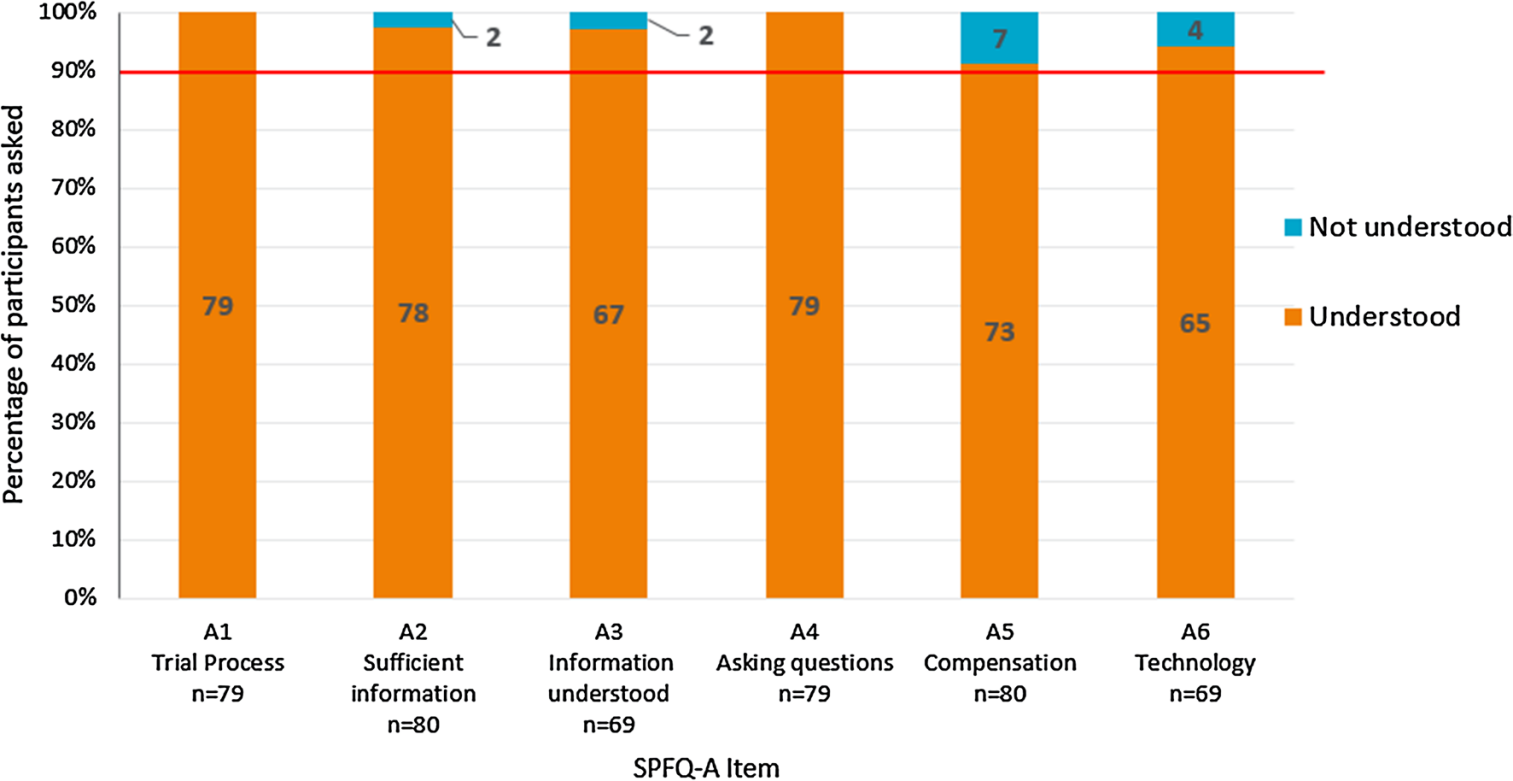
Summary of Understanding of SPFQ-A Items. Red line indicates 90% of the sample. Items were considered potentially problematic if less than 90% of participants understood the items or thought that they were important.

Participants’ understanding and interpretation of the SPFQ-A items was high, with over 90% of the study participants demonstrating understanding and accurate interpretation of all six items (Fig. 1). In line with this, the response options of the SPFQ-A were consistently well understood.

**Figure 2:**
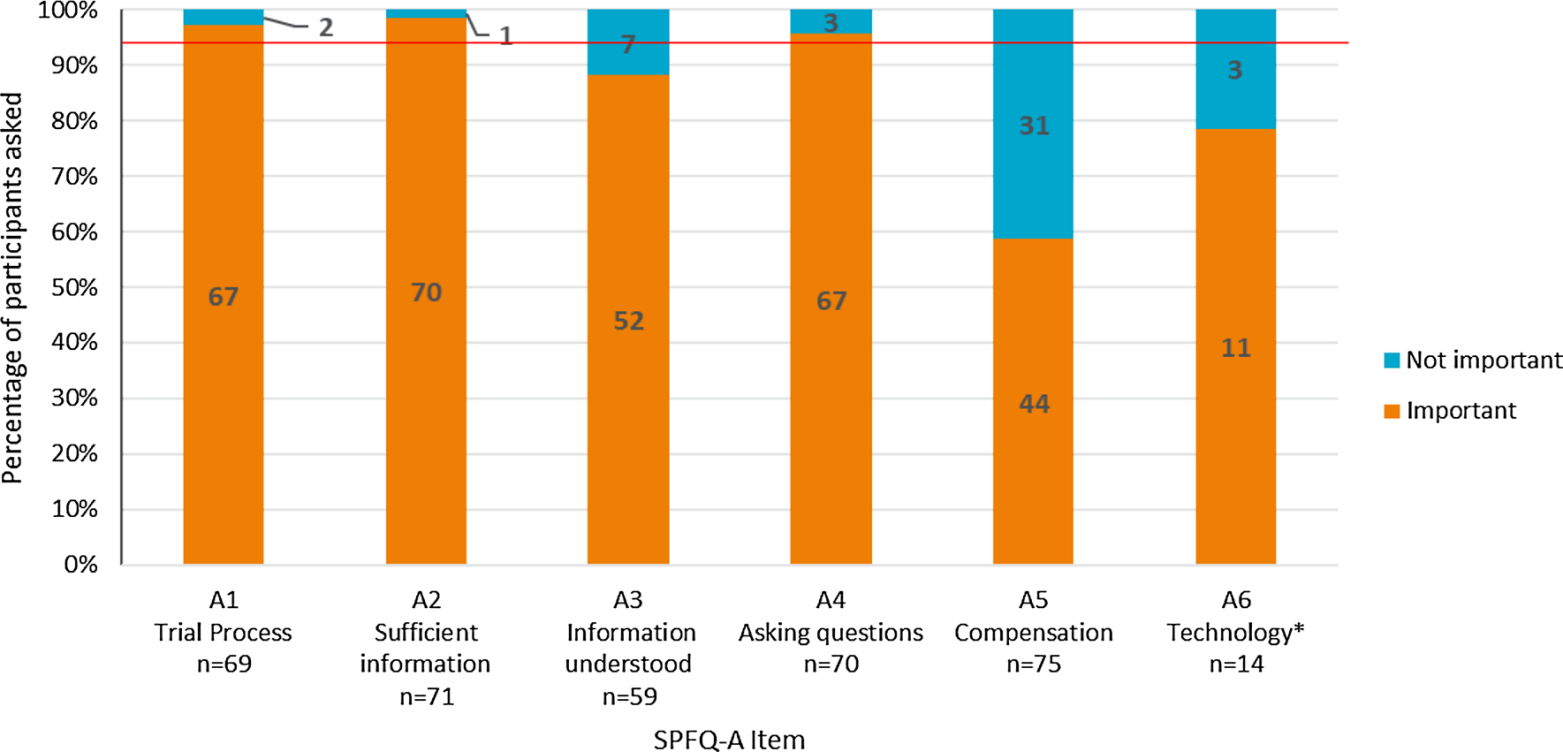
Summary of Importance of SPFQ-A Items. Red line indicates 90% of the sample. Items were considered potentially problematic if less than 90% of participants understood the items or thought that they were important.

Participants indicated that half of SPFQ-A items were important to their clinical trial experience, with over 90% indicating importance for items A1 (understanding treatment process), A2 (information received prior to trial) and A4 (asking questions about the trial) (Fig. 2). However, some participants did not consider item A3 (ability to understand information given before trial) to be important to their trial experience (*n* = 7/59; 11.9%), with five participants suggesting conceptual overlap with item A2 (*n* = 5/8; 62.5%). The perceived importance of item A5 (understanding of compensation) was also relatively low, with most participants (*n* = 18/31; 58.1%) indicating that they did not receive any compensation. Three participants also did not consider item A6 (use of electronic questionnaires during the trial) to be important (*n* = 3/14; 21.4%), indicating that electronic questionnaire completion was secondary to their satisfaction with the trial, and was therefore not important to include (*n* = 3/3; 100.0%).

Feedback on the SPFQ-A items was mostly consistent at the subgroup level. The only item which demonstrated cross-country differences was item A4, which assesses the extent to which participants feel comfortable to ask questions. Three of the participants that believed this item should be reworded (specifically the rewording of ‘comfortable’ to ‘safe’ or ‘absolutely sure’) were German (*n* = 3/4; 75.0%). Nonetheless, all German participants understood the item and were able to provide responses (*n* = 10/10; 100.0%).

#### SPFQ-B

**Figure 3:**
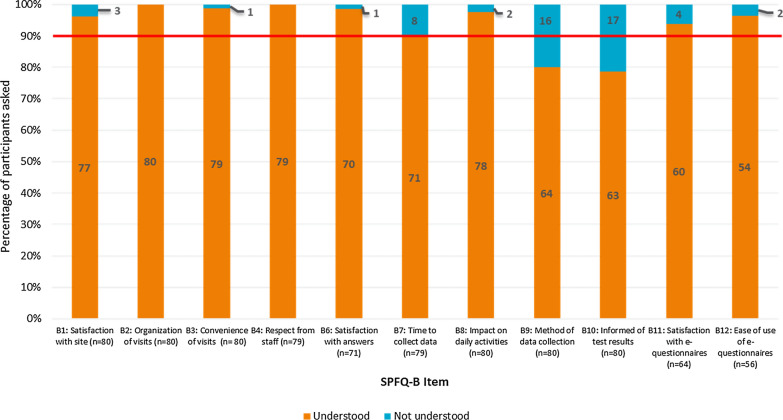
Summary of Understanding of SPFQ-B Items. Red line indicates 90% of the sample. Items were considered potentially problematic if less than 90% of participants understood the items or thought that they were important.

Participants’ understanding of the SPFQ-B items was generally high with over 90% of the study participants demonstrating an understanding and accurate interpretation of nine of the 12 items. However, issues with understanding and interpretation were apparent for three of the items (Fig. 3). Eight participants had difficulty understanding item B7 (acceptability of time taken to collect data), with difficulties focused on the word ‘data’ (*n* = 8/79; 10.1%). A number of participants also experienced difficulties understanding item B9 (methods for data collection) (*n* = 16/80; 20.0%), particularly the wording of ‘wearable sensors’, ‘monitoring devices’, and ‘diary’ in parenthesis. Seventeen participants also experienced difficulties understanding item B10 (feedback of medical test results), particularly the word ‘screening’ (*n* = 17/80; 21.3%). Of note, items B9 and B10 are both optional items of the SPFQ only intended for administration in trials where appropriate. As such, issues related to understanding and interpretation of certain items may be a result of some participants not having direct experience of these aspects of a clinical trial and therefore lacking familiarity (i.e. participants may not have used wearable sensors and therefore did not understand the term). The response options of the SPFQ-B were consistently well understood.

**Figure 4:**
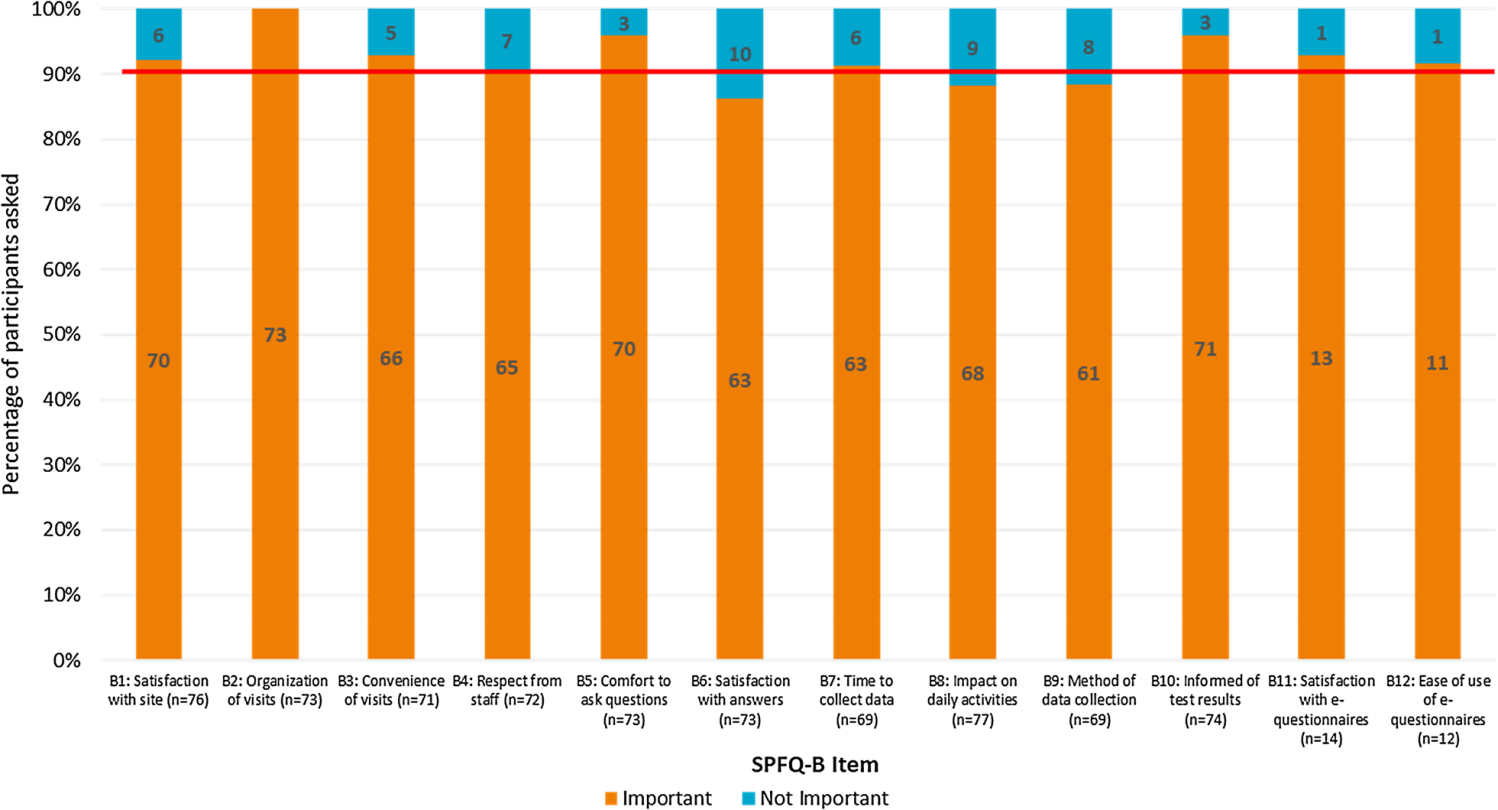
Summary of Importance of SPFQ-B Items. Red line indicates 90% of the sample. Items were considered potentially problematic if less than 90% of participants understood the items or thought that they were importance.

Participants indicated that the majority of the items of the SPFQ-B were important to assess (Fig. 4). However, seven participants (*n* = 7/72; 9.7%) reported that item B4 (assessing staff attitudes) was not important, indicating that respectful staff is implicit to a clinical trial, and therefore not important to assess. Ten participants also did not consider item B6 (assessing answers to questions) to be important (*n* = 10/73; 13.7%), explaining that they did not ask questions during the trial and that the items overlapped conceptually with item B5 (assessing feeling comfortable to ask questions). A number of participants also reported that item B8 (assessing impact on daily activities) was not important (*n* = 9/77; 11.7%), reporting that the trial had no impact on their daily activities. Additionally, a total of eight participants did not consider item B9 (relating to data collection methods) to be important (*n* = 8/69; 11.6%), explaining that they either felt indifferent about data collection methods or had not experienced any of the data collection methods detailed in the parenthesis.

When assessing understanding and perceived importance of SPFQ-B items on a subgroup level, findings were consistent across the groups.

#### SPFQ-C

**Figure 5:**
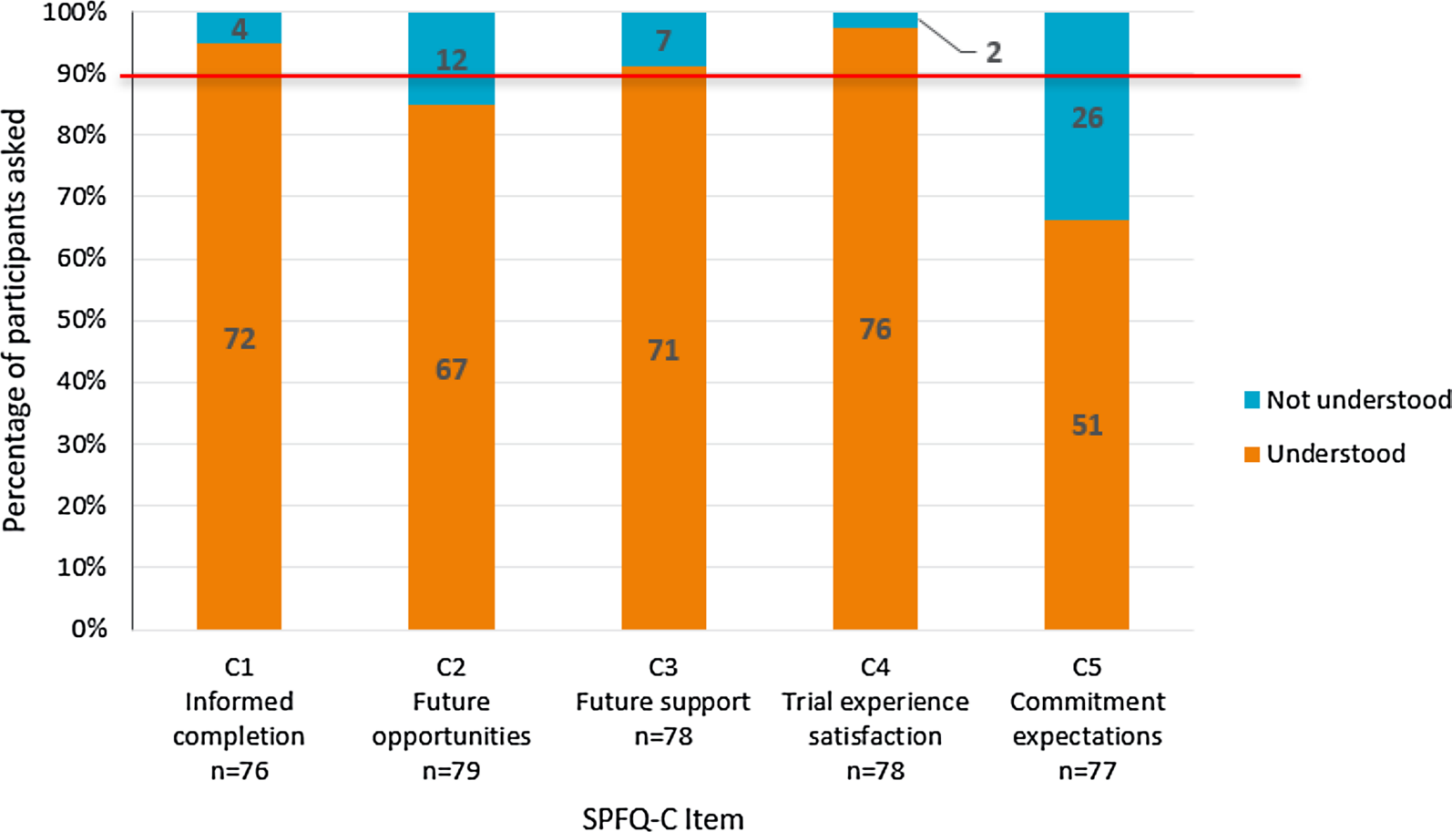
Summary of Understanding of SPFQ-C Items. Red line indicates 90% of the sample. Items were considered potentially problematic if less than 90% of participants understood the items or thought that they were important.

Participants’ understanding and interpretation of the majority of the SPFQ-C items was high, with over 90% of the sample demonstrating understanding and accurate interpretation of most items (Fig. 5). However, twelve participants (*n* = 12/79; 15.2%) demonstrated difficulty understanding item C2 (assessing opportunities to access trial results), particularly the wording of ‘future opportunities’. Additionally, 26 participants (*n* = 26/77; 33.8%) had difficulty understanding item C5 (assessing overall commitment for trial), misinterpreting the item to refer to commitment from the site staff, rather than their own commitment. Response options of the SPFQ-C were consistently well-understood.

**Figure 6:**
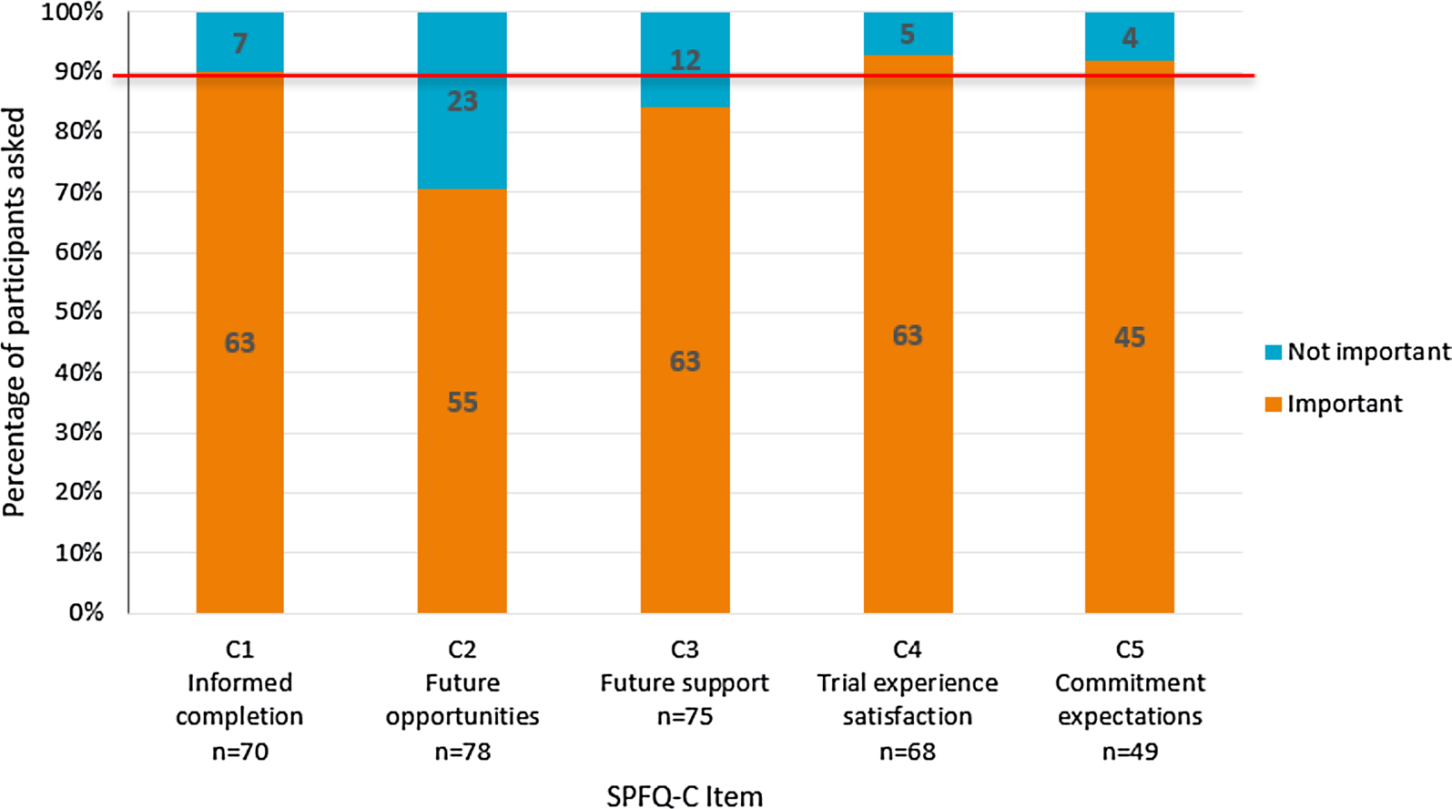
Summary of Importance of SPFQ-C Items. Red line indicates 90% of the sample. Items were considered potentially problematic if less than 90% of participants understood the items or thought that they were important.

Participants indicated that the majority of the items of the SPFQ-C were important to assess (Fig. 6). However, a number of participants (*n* = 23/78; 29.5%) did not consider item C2 (assessing opportunities to access trial results) to be important, reporting that they were not given opportunities to access the overall trial results. Additionally, 12 participants (*n* = 12/75; 16.0%) indicated that item C3 (assessing future support after the trial) was not important to assess, reporting that they did not receive any information about future support.

Feedback on the SPFQ-C items was mostly consistent at the subgroup level. Country differences were only apparent for item C3 (which asks about future support). It was found that five of the participants who did not receive future support were from China (*n* = 5/12; 41.7%).

### General Feedback

The instructions of the SPFQ, which are consistent across all three questionnaires, were also debriefed as part of the online version of the questionnaire and were well understood by most participants (*n* = 67/72; 93.1%). The recall period was also well understood consistently across the SPFQ-A (*n* = 42/47; 89.4%), SPFQ-B (*n* = 42/46; 91.3%) and SPFQ-C (*n* = 38/41; 92.7%). Participants had very few suggestions for items that should be added to the SPFQ. The most frequently reported missing concept was side-effects (SPFQ-B: *n* = 3/12; 25.0%, SPFQ-C: *n* = 2/15; 13.3%), however this information is typically collected via safety reporting during a trial and is considered a feature of the intervention under investigation rather than the design and procedures of the clinical trial. Across all three of the questionnaires, participants explained that they liked that the SPFQ was clear, relevant and comprehensive. However, a small proportion of participants (*n* = 12/80; 15.0%) felt that some items were repetitive or similar in content (particularly in the SPFQ-A: *n* = 5/12; 41.7%). Participants also reported that the questionnaires were an appropriate length, and not burdensome to complete; SPFQ-A (*n* = 72/77; 93.5%), SPFQ-B (60/70; 85.7%) and SPFQ-C (*n* = 68/72; 94.4%). Overall, all three of the questionnaires were reported as being easy to complete, SPFQ-A (*n* = 68/70; 97.1%), SPFQ-B (*n* = 56/56; 100.0%) and SPFQ-C (*n* = 58/59; 98.3%).

## Discussion

The complexity of many clinical trial designs, and the information provided to participants, create barriers to participant recruitment and retention [20]. In an effort to increase the efficiency of trial processes, it is essential to take a patient-centric approach. Obtaining feedback from clinical trial participants can identify aspects of trial design that could be improved, which may help to increase participant compliance and retention. However, few sponsors of clinical studies have consistent, company-wide processes for collecting feedback from study participants [21]. Developed in accordance with best practices for the validation of PRO instruments [3, 13], the SPFQ was designed as a measure of participant satisfaction and understanding of clinical trial procedures for use in a range of indications and trial designs [11, 12]. This study aimed to evaluate the content validity and cross-cultural validity of the SPFQ.

Concept elicitation findings support the content validity of the SPFQ, confirming that concepts measured by the SPFQ reflect those considered most important to participants spontaneously providing feedback regarding their experiences participating in clinical studies. Participant reports of motivations for taking part in clinical trials, the benefits and disadvantages of trial participation, and opportunities to improve future trial participation were consistent to those reported in existing literature [22–25].

Findings from the cognitive debriefing section of the interviews indicated generally high levels of understanding, interpretation and perceived importance across items, supporting the content validity of the SPFQ. Instructions, recall periods and response options were also generally well understood. However, it is important to acknowledge that feedback from participants in relation to a select number of items highlighted potential issues regarding comprehension, suggesting that further investigation and potential revision of these particular items may be warranted. For example, item C5 (commitment expectations) was understood and accurately interpreted by only 66.2% of the sample, but 91.8% of the sample emphasised the importance of this particular concept in the context of evaluating trial satisfaction. Completion of the SPFQ as a formal trial procedure (rather than a retrospective evaluation as in the current study) may alleviate some of the issues identified. An implementation user guide has also been developed to support sponsor implementation of the SPFQ in clinical studies (e.g. objectives of the SPFQ and instructions for completion) that may be used to facilitate administration and improve clarity (https://transceleratebiopharmainc.com/patientexperience/study-participant-feedback-questionnaire/). Nonetheless, however, depending on the context in which the SPFQ is to be used (trial design and/or study population), investigators may wish to consider the refinement of specific items such as C5 (e.g. the addition of examples or use of alternative terminology) for future applications.

The SPFQ is intended as a generic measure for use across various clinical study designs, and a wide range of therapeutic indications. As a result, there are a number of items in the SPFQ which are designed for modular components for inclusion in the measure, to be implemented in studies as appropriate. For the purposes of this particular study, all items were tested with participants which led to the identification of items considered not to be important by a subset of participants. For example, a large proportion of the sample had not received compensation or used electronic technology in their trial. As a consequence, the number of participants that could provide feedback on the perceived importance of the items or considered this item important was limited. It is important, therefore, for sponsors to consider which of those items of the SPFQ are suitable for administration in the context of the respective study protocol and target patient population.

The SPFQ has been translated and linguistically validated for use in a wide number of languages. The findings from this study further provide evidence for cross-cultural validity since few differences in understanding and perceived importance of items were observed between participants from different countries. Similarly, consistent understanding and importance of items found across patients in different disease areas supports the use of the SPFQ as a generic measure across therapeutic indications. As the SPFQ was originally developed for use primarily in an oncology setting [11, 12], these findings are valuable when considering possible future applications of the measure. Furthermore, participants provided positive feedback regarding ease of completion of the web-based version of the SPFQ. When considering future applications, these findings support the utility of an electronic version of the SPFQ. The use of electronic instruments in clinical trials is beneficial in terms of deployment and collection of data [26], particularly when implemented globally.

One of the key benefits of the SPFQ is the fact that the instrument is divided into three questionnaires to be completed at key stages during the clinical trial. The benefit of implementing multiple questionnaires across different timepoints (as opposed to just at the end of the trial) is that sponsors may be able to identify and address problematic issues as the trial is ongoing, which could enhance clinical trial experience and increase the likelihood of participant compliance and retention [27, 28].

Rates of discontinuation and attrition are significant challenges for clinical trials adversely impact and bias outcomes. It is difficult to estimate rates of discontinuation in clinical trials due to inconsistencies in reporting. There are likely a number of trial-specific factors that contribute to participant discontinuation and retention. However, it is estimated that approximately 7–11% of participants randomised to receive treatment may discontinue trials early [29, 30]. In appreciation that the experiences of participants discontinuing early from clinical trials may be distinct from those completing clinical trials, efforts were made to recruit a proportion of trial withdrawers for this study (*n* = 10/80; 12.5%). However, challenges were encountered in identifying such participants (the reasons for which are unclear), meaning that representation of these participants in the study sample was less than originally targeted (*n* = 6/80; 7.5%). Feedback from those participants who had discontinued a clinical trial suggests that items included in the SPFQ are sufficient for providing insight into reasons for trial withdrawal. Nonetheless, the failure to recruit more withdrawers may be considered a limitation of the current study, and further exploration of validity of the SPFQ among this specific population would be valuable.

Additionally, information was collected through a participant-completed screener. While most participants were able to provide details about the trial in which they participated (e.g. therapeutic area, clinical trial phase, investigational product), this information wasn’t always available. Efforts were made to try and identify further details (e.g. asking participants to specify the name of the trial) to complete this information but it was not always possible. As such the inability to compare and contrast the experiences of participants by some parameters (e.g. clinical trial phase), and the fact that some participants may have been completing items assessing concepts that are not applicable to the trial in which they participated, is acknowledged as a limitation of the study.

Finally, further limitations of the design were the requirement for the SPFQ to be read and completed online (ruling out participation for those with very low health/digital literacy or no access to the internet) and the fact that participants were reflecting on their historical experiences of participation in a clinical trial (as much 3 years ago). It is possible that the feedback may not reflect thoughts and opinions at the time of participation. Furthermore, it is possible that participants completing all questionnaires following completion of the trial (when SPFQ-A and SPFQ-B are to be completed prior to and during the commencement of the trial) may have contributed to some of the observed issues.

## Conclusion

This is the first study to report on a global measure of clinical trial experience. Overall, the findings from this study provide support for the cross-cultural validity of the SPFQ and support the utility of including the SPFQ as an exploratory outcome measure in clinical trials to obtain clinical trial feedback and inform future trial design, regardless of the therapeutic indication or country. As one of the first steps in the development and validation of the SPFQ, it is recognised that further refinement and evaluation may be necessary depending on the trial design or therapeutic area to optimise comprehension and the perceived importance of items. Implementation of the SPFQ in future global clinical trials across a range of therapeutic indications is expected to provide useful insights into patient perspectives of clinical trial participation. Such evidence will also be critical for facilitating the continued development and refinement of the SPFQ with the goal of helping to incorporate the patient voice in drug development and evaluation.

## Electronic supplementary material

Below is the link to the electronic supplementary material.

Supplementary file1 (DOCX 371 kb)
