# Supplementary material for: Evaluation of the Content Validity and Cross-Cultural Validity of the Study Participant Feedback Questionnaire (SPFQ)
Source: Ther Innov Regul Sci. 2020 Jul 20;54(6):1522–33. doi: 10.1007/s43441-020-00179-3 (PMC7704515; doi:10.1007/s43441-020-00179-3)

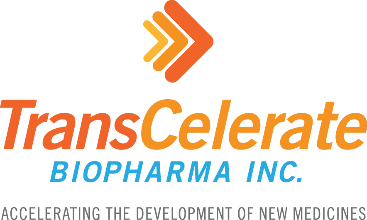


**Patient Experience Initiative**

Study Participant Feedback Questionnaire (SPFQ)

**Version 1.0**

**Prepared by:**

TransCelerate Patient Experience Initiative Team

This deliverable prepared by TransCelerate BioPharma can be adopted by member companies and others, but all adoption is purely voluntary and based solely on the particular company’s unilateral decision. TransCelerate has provided this Study Participant Feedback Questionnaire (“SPFQ”) and the corresponding User Guide (collectively the “Work Product”) for informational purposes only. By using the Work Product, you manifest your assent to the terms of use set out in this paragraph. The Work Product are not tailored to any particular factual situation and are provided ‘AS IS’ WITHOUT WARRANTY OF ANY KIND, EITHER EXPRESSED OR IMPLIED, INCLUDING, BUT NOT LIMITED TO, THE IMPLIED WARRANTIES OF FITNESS FOR A PARTICULAR PURPOSE, NON-INFRINGEMENT, OR MERCHANTABILITY. TransCelerate and its members do not accept any responsibility for any loss of any kind including loss of revenue, business, anticipated savings or profits, loss of goodwill or data, or for any indirect or consequential loss whatsoever to any person using the Work Product. Any party using the Work Product bears sole and complete responsibility for ensuring that the Work Product, whether modified or not, are suitable for the particular clinical trial, accurate, current, commercially reasonable under the circumstances, and comply with all applicable laws and regulations.

# Section A: Your experience before you started the study <to be completed within 1 month of study enrollment>

*Thank you for your participation. Your experiences in this trial are important to us and we would like to hear about them. Your answers will help us improve future trials. There are no right or wrong answers, and it will take approximately 15 minutes to complete. Your answers will be kept anonymous and will not impact your participation in this trial.*


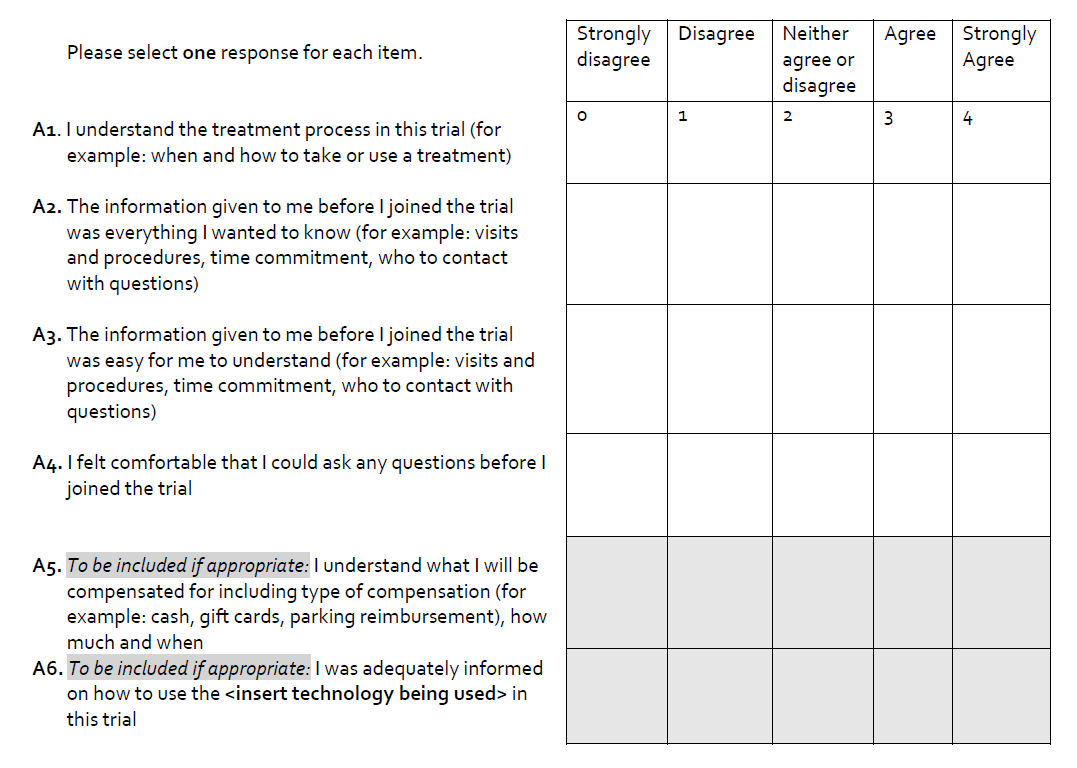


# Section B: Your experience during the trial <to be completed during trial progress>

*Thank you for your participation. Your experiences in this trial are important to us and we would like to hear about them. Your answers will help us improve future trials. There are no right or wrong answers, and it will take approximately 15 minutes to complete. Your answers will be kept anonymous and will not impact your participation in this trial.*


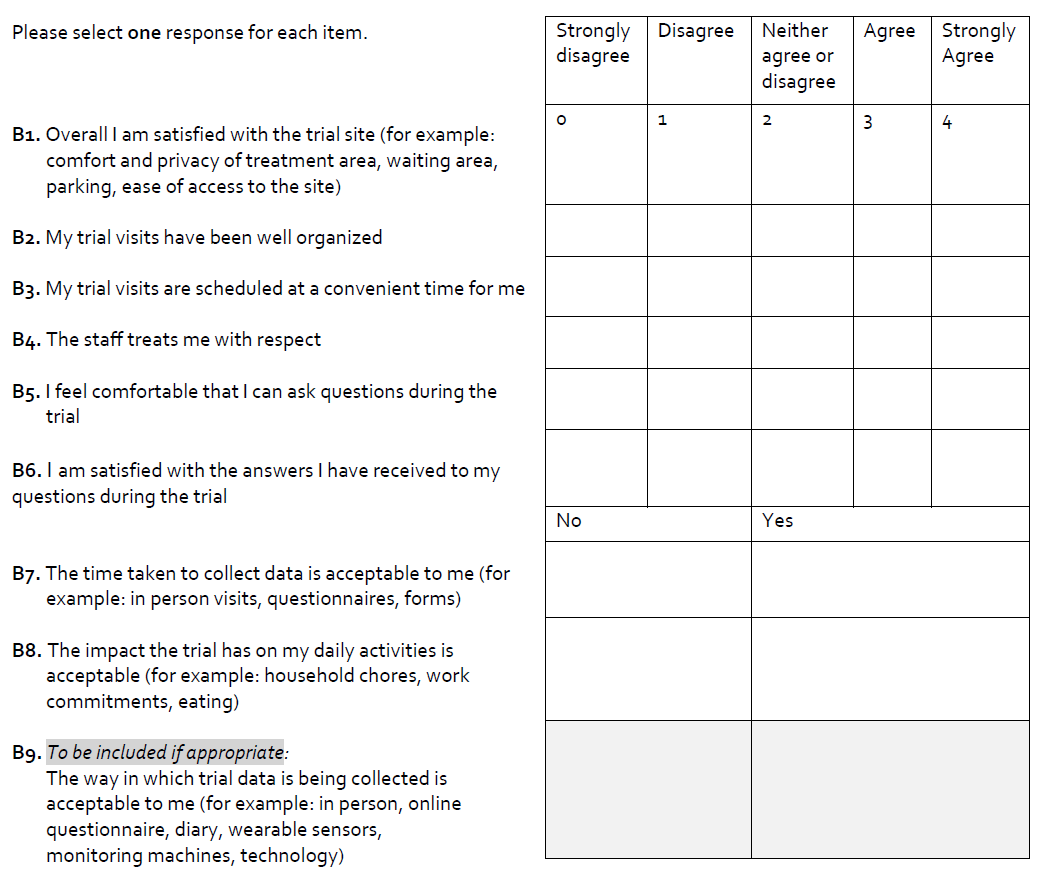


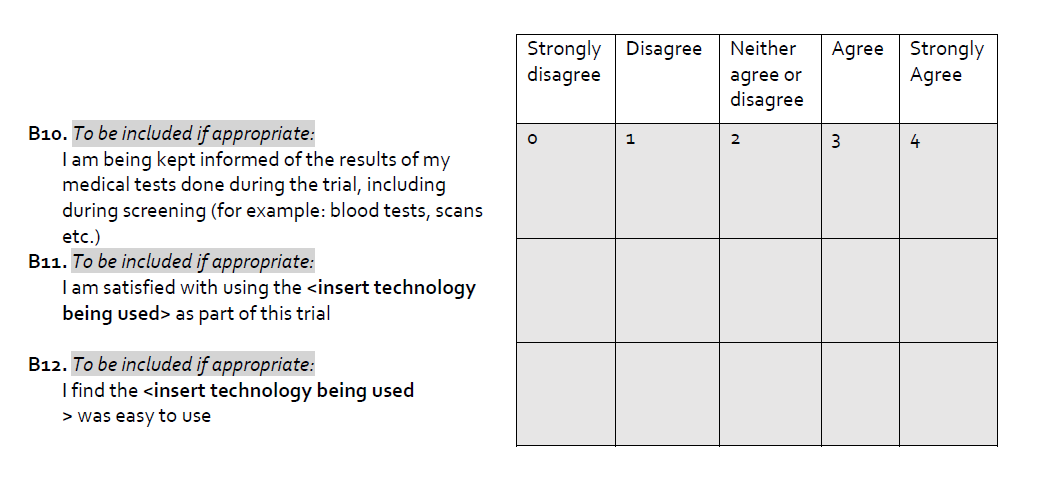


# Section C: Your experience at the end of the trial <to be completed at last trial visit>

*Thank you for your participation. Your experiences in this trial are important to us and we would like to hear about them. Your answers will help us improve future trials. There are no right or wrong answers, and it will take approximately 15 minutes to complete. Your answers will be kept anonymous and will not impact your participation in this trial.*


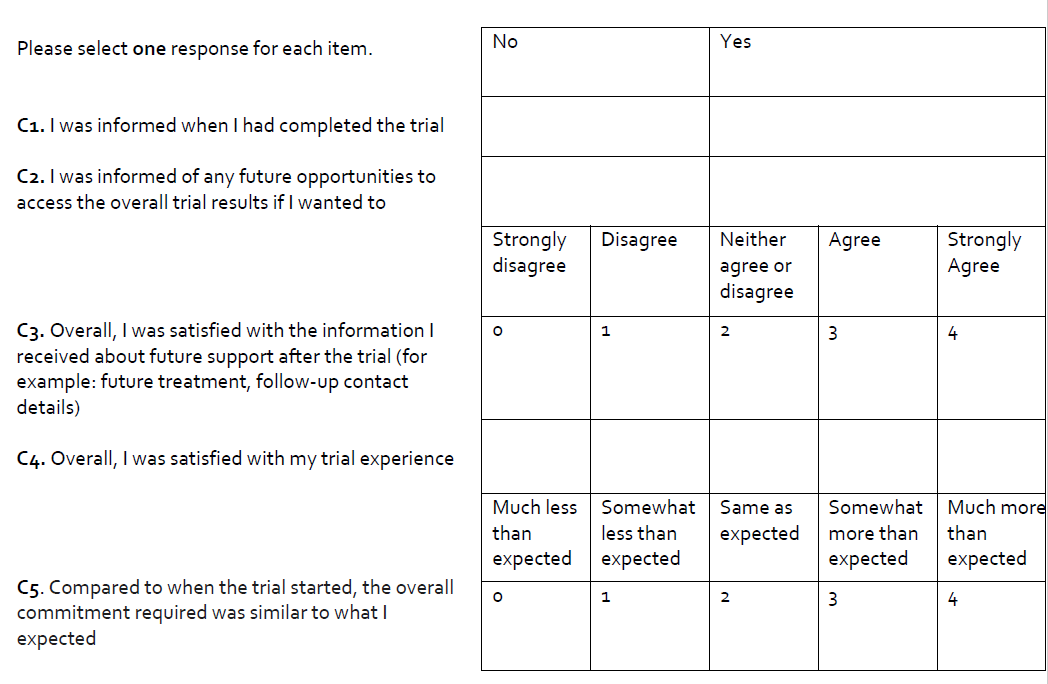

Supplement: Supplementary file 1 — Supplementary file1 (DOCX 371 kb) [file 43441_2020_179_MOESM1_ESM.docx]
